# Erlotinib and Concurrent Chemoradiation in Pretreated NSCLC Patients: Radiobiological Basis and Clinical Results

**DOI:** 10.1155/2013/403869

**Published:** 2013-08-04

**Authors:** Sara Ramella, Antonio Maria Alberti, Eugenio Cammilluzzi, Michele Fiore, Edy Ippolito, Carlo Greco, Angelo Luca De Quarto, Sara Ramponi, Giovanni Apolone, Lucio Trodella, Alfredo Cesario, Rolando Maria D'Angelillo

**Affiliations:** ^1^Radiation Oncology, Campus Bio-Medico University, Via Álvaro del Portillo 21, 00128 Roma, Italy; ^2^Medical Oncology, Sandro Pertini Hospital, Via dei Monti Tiburtini 385, 00157 Roma, Italy; ^3^Scientific Directorate, IRCCS-Arcispedale Santa Maria Nuova, Viale Risorgimento 80, 42123 Reggio Emilia, Italy; ^4^Thoracic Surgery, Catholic University, Largo Agostino Gemelli 8, 00168 Roma, Italy; ^5^Deputy Scientific Director, IRCCS San Raffaele Pisana, Via della Pisana 235, 00163 Roma, Italy

## Abstract

*Aims*. To establish feasibility of the combination of Erlotinib and concurrent chemoradiation in pre-treated patients with locally advanced or metastatic NSCLC. *Materials and Methods.* Data regarding 60 consecutive patients with NSCLC previously treated with chemotherapy alone were prospectically collected. All patients started Erlotinib concurrently with chemotherapy and radiation delivered to primary tumor. These data were retrospectively analyzed (observational study). Feasibility and toxicity were the primary endpoints, with response rate and progression being the secondary ones, while survival data are reported just as exploratory analysis. The EGFR mutational status was recorded in 32% of cases and it was always wild type. *Results*. Compliance to the combination protocol was good. Grade 3-4 esophagitis and acute lung toxicity occurred in 2% and 8% of patients, respectively. No progressive disease was recorded in the majority of cases (65%). Median OS and PFS were 23.3 and 4.7 months, respectively. Patients not responding to chemotherapy administered prior to chemoradiation achieved an objective response rate of 53.3% and complete response in 13.3% of cases. *Conclusions*. The addition of Erlotinib to chemoradiation in inoperable NSCLCs is feasible with interesting efficacy profile. These preliminary results warrant further investigation in patients with locally advanced nonmetastatic NSCLC with EGFR mutations.

## 1. Introduction

The overexpression of EGFR plays a key role in cellular proliferation, metastasis, apoptosis inhibition, and chemoradioresistance [1]; its targeting with tyrosine kinase inhibitors (TKIs) has improved survival in metastatic NSCLC patients [2]. The standard treatment for locally advanced inoperable NSCLC is concurrent chemoradiation. With modern techniques, it guarantees effective and safe treatment [3]. Experimental evidence suggests that the TKIs could have a radiosensitizing effect [4]. Many mechanisms of this radiosensitizing effect have been proposed and in vivo studies have confirmed synergistic growth inhibition of radiation and TKIs [5]. In NSCLC models Erlotinib clearly enhances radiation cytotoxicity [6]. Radiobiological criteria and basis of evidence have led to investigating the role of this combination at several levels.

- Radiosensitization of the cancer cell by altering intracellular signaling: as previously reported, the overexpression of EGFR in solid tumors is correlated with increased radioresistance. This is apparently due, in first instance, to the ability of radiation to interact with EGFR causing receptor activation even in absence of EGF, for instance by TGF*α* release and EGFR autophosphorylation increase. This activation triggers intracellular pathways cascade, mainly via the RAS/RAF/MAP kinases (resulting in proliferative stimulus) and via the PI3K/AKT one (resulting in the inhibition of the apoptosis). This effect has been proposed to represent a central mechanism for accelerated cellular repopulation during radiation treatment [7].
- Cell cycle kinetics: Erlotinib, when combined with radiation, has been demonstrated to be able to promote a reduction in the S-phase fraction (which is the most radioresistant cell cycle phase), inducing accumulation of cells in G1 and G2 [8].
- Apoptosis: Erlotinib and radiation induced an increase in apoptosis as determined by caspase activity. Poly(ADP-ribose) polymerase (PARP) cleavage increases when Erlotinib is combined with radiation [8].
- DNA repair: Erlotinib attenuates radiation induced expression of DNA repair protein [8]. The main action of radiation is killing cancer cells by DNA damage. When radiation reaches cell surface causes EGFR internalization. The receptor moves into the nucleus by binding proteins (Ku70/Ku80 and DNA-PKcs) and activates damage repair. If EGFR is blocked by antibodies or TKIs, the complex does not enter the nucleus resulting in inhibition of DNA repair. Rad51 is a repair protein which represents a central part of the homologous recombination process during the DNA repair and Erlotinib attenuates the increase of Rad51 after radiation exposure [9]. The potential influence of Erlotinib on the DNA damage repair is amplified in vivo respect to in vitro setting because of the delivering of multiple versus single fractions of radiation [8].
- Clonogenic cells: Erlotinib influences cancer cell clonogenic survival, with a modest but consistent reduction in clonogenic survival when the TKI is administered before the radiation treatment [9].

To the best of our knowledge, only sporadic reports exist in the literature about clinical experiences adopting this combination which refer to small populations treated with small molecules plus radiation, with or without concurrent chemotherapy [10–12].

Currently, Erlotinib has a well-established role in first- and second-line treatment but few data are still present in locally advanced NSCLC while radiotherapy has shown an important action against symptoms onset. Sometimes, in daily clinical practice, patients treated with upfront chemotherapy experienced minimal response with stable disease or partial response with tumor reduction among 30% in volume and are candidates for palliative chemoradiation or second line Erlotinib. The aim of this study was twofold: to report feasibility (defined as compliance to the protocol) and activity of combining both strategies (Erlotinib plus chemoradiation) in this poor prognostic group.

## 2. Materials and Methods

We treated patients with locally advanced (IIIA-IIIB) NSCLC (excluding those with cytologically confirmed malignant pleural or pericardial effusion) or mediastinal recurrences after surgery or oligometastatic disease (up to two sites of distant metastasis) with good performance status and adequate blood marrow reserve. All patients received upfront chemotherapy and those classified as nonresponders or minimal responders (among 30% in reduction of tumor volume) went on to have Erlotinib 150 mg PO daily, delivered concurrently with chemoradiation.

Induction chemotherapy consisted in 4–6 cycles of chemotherapy according to patient's tolerance and the revaluation CT was performed about 1 month after the last chemotherapy cycle.

Radiotherapy was delivered using a linear accelerator (CLINAC C2100, Varian) with a 6–15 MV photon beam up to a median dose of 59.4 Gy and 1.8 Gy as daily fractionation. All patients underwent 3D-treatment planning and were immobilized by customized devices. Radiotherapy was administered with an angled fields technique (planar and no planar) [13] to include the Planning Target Volume (PTV) in the 95–107% isodose area. The Gross Tumor Volume (GTV) was defined as tumor extension and metastatic lymph nodes CT and/or PET 18FDG defined. The Clinical Target Volume was equal to GTV, and the PTV consisted of GTV plus 1 cm margin. Elective Nodal Irradiation was never used. The dose-volume constraints for total lung were set as follows: V20 ≤ 31%, V30 ≤ 18%, and MLD ≤ 20 Gy; for the ipsilateral lung V20 ≤ 52% and V30 ≤ 39%; the maximum spinal cord dose was 38 Gy; for the esophagus V50 < 30%, and for the heart V40 < 50%. 

The total dose of 59.4 Gy was delivered in 33 fractions/6.5 weeks of treatment duration. Seven weekly gemcitabine (350 mg/mq) and two pemetrexed cycles (500 mg/mq) were administered concurrently to radiotherapy.

Toxicity was recorded according to the Common Toxicity Criteria scale 3.0 (CTC-AE). When grade 2 or 3 esophageal, pulmonary, and cardiac toxicity or grade 3-4 hematological and skin toxicity appeared, radiation was interrupted and restarted upon resolution. Administration of chemotherapy was delayed in case of grade 2 hematological toxicity, while a 25% dose reduction was applied if grade 3 hematological toxicity appeared. Radiotherapy was discontinued in case of grade 4 nonhematological toxicity or persistent grade 3 nonhematological toxicity with symptoms not recovered after 14 days with specific therapy. Chemotherapy was discontinued in the case of grade 4 hematological toxicity. 

Response evaluation was performed using the Response Evaluation Criteria in Solid Tumor (RECIST) by CT scan 4-5 weeks after the end of the treatment. 18 FDG PET/TC was performed after 4–6 months after the end of radiotherapy, then annually or for clinical suspicious.

Pyrosequencing was performed on material obtained from histological assessment, to verify the sequencing data of the hotspots of EGFR (exons 18-19-21) and to assess the proportion of mutant alleles in microdissected specimens using a Pyrosequencing PSQ 96MA (Pyrosequencing, Uppsala, Sweden). Patients underwent a monthly followup for the first 3 months every 3 months for the following 2 years, and every 4–6 months for the next 3 years.

### 2.1. Statistical Analysis

Primary endpoints were feasibility and toxicity. The secondary endpoints included response rate and progression. Overall survival (OS; defined as the time between diagnosis and event or last visit) and progression-free survival (PFS; defined as the time between the end of chemoradiation and disease progression) were reported only as exploratory data. Statistical analysis was performed with SYSTAT, ver. 11.0 (SPSS, Chicago, IL).

## 3. Results

### 3.1. Patients Characteristics

Between July 2007 and May 2010, 60 consecutive patients were observed and treated. Patient's characteristics are listed in Table 1. Median age was 65 years (range 39–83), with male prevalence (70%). Twenty patients had a stage IIIA disease (33%), 19 had stage IIIB (32%), 6 had mediastinal recurrences after surgery (10%), and 15 patients had oligometastatic disease (25%). Among metastatic patients, 14 had one distant metastasis while the last one had one lung and one brain metastases (total distant metastases: 16). Histology was nonsquamous in 65% of cases, squamous in 33%, and not otherwise specified in 1 patient (2%). 

Fift-four patients (90%) received upfront chemotherapy, whilst the remaining 6 patients were postsurgery, with median number of 4 courses (range 1–12). In 28 patients (52%) chemotherapy obtained a stable disease, while 24 and 2 patients had a minimal partial response and a progression disease, respectively. 

### 3.2. Toxicity

Table 2 summarizes treatment related toxicities. Only one patient was unable to complete the combined treatment as planned due to toxicity (pulmonary), while two patients stopped treatment due to tumor progression and one due to a fall in performance status. Four patients (7%) developed a grade 3 skin rash requiring dose reduction of Erlotinib to 100 mg daily. Grade 3 diarrhea occurred in 2 patients while nausea, mucositis, and photophobia were reported in a few patients and always classified as grade 1-2 toxicity. Grade 3-4 esophagitis and pulmonary toxicity occurred in 2% and 8% of patients, respectively. After treatment end, two patients experienced a fatal pulmonary toxicity: one experienced radiation pneumonitis associated with multiple pulmonary metastasis, while the other one developed sepsis with positive hemoculture for *Staphylococcus epidermidis*. Subacute and late pulmonary toxicity was recorded in 23% of patients as radiographic changes not requiring oxygen therapy. Finally, grade 3-4 hematological toxicity was recorded as follows: thrombocytopenia 12%; leucopenia 30%; anemia 5%. Liver toxicity, recorded as modification in liver enzymes, occurred as grade 3-4 toxicity in 8 patients, requiring Erlotinib and chemotherapy end.

**Figure 1:**
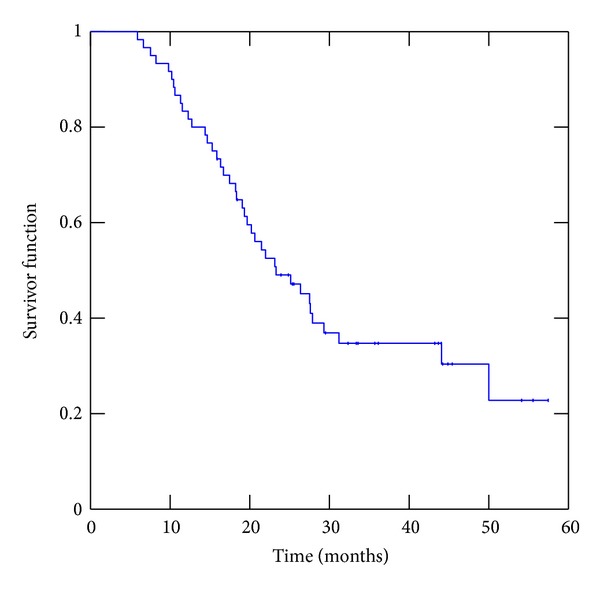
Overall survival for the whole group.

Complete or partial response, and stable disease were achieved in 6, 18, and 15 patients, respectively, accounting for an overall 65% of “no progressive disease.” Three patients had an in-field progression (5%), 1 had local and distant relapse, and 17 showed distant relapse (28%). Patients who are “no responders” to upfront chemotherapy, achieved an objective response rate of 53.3% after chemoradiation and TKIs combination with “complete response” in 13.3% of cases. No particular association was noted between skin rash or histology with clinical response. Survival median survival was 23.3 months (Figure 1). One-, 2-, and 3-year overall survival was 81%, 46%, and 29%, respectively.

**Figure 2:**
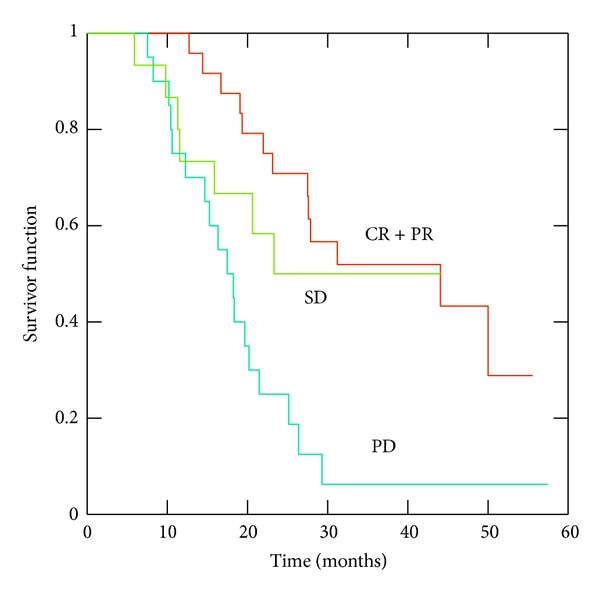
Overall survival according to clinical response after concurrent treatment with chemoradiation and TKIs therapy (*P* = 0.001). Legend: CR: complete response; PR: partial response; SD: stable disease; PD: progression disease.

As expected, patients with complete/partial response experienced a better survival (44 months) than no-responder ones (23.3 months) or those with progressive disease (17.5 months; *P* = 0.025) (Figure 2). With a median followup from the end of concomitant therapy of 33.7 months, we have recorded 39 deaths, while 14 patients are still alive with residual tumor and 5 patients are free from any tumor evidence. Analysis of the EGFR mutation status was performed in 19/60 patients (32%); none of these had EGFR mutations.

## 4. Discussion

Our population includes pre-treated patients with poor response after front-line chemotherapy. In this poor prognostic group, our results appear of interest with a median overall survival of 23.3 months, an objective response rate of 53.3%, and complete response in 13.3% of cases.

Toxicity profile revealed a slightly high pulmonary toxicity. In general, the treatment with TKIs and concurrent radiation without chemotherapy showed a 4% of G3-4 lung toxicity [12]. It is well known that adding chemotherapy to radiation causes an improvement in survival at the price of a higher toxicity. The grade 3 pneumonitis for patients treated with concurrent EGFR-TKI and chemoradiation is historically in the range of 6–8% [11]. Reporting about the outcome of a phase I trial where gefitinib was administered concurrently with chemoradiation and used alone as maintenance therapy, G3-4 lung toxicity was 20% [12]. In our study the incidence of G3-5 lung toxicity is 8% and late pulmonary fibrosis was recorded only as G1-2 toxicity grade in 23% of patients; this is in line with toxicity levels of standard chemoradiation protocols. In recently reported phase III concomitant chemoradiation trials for locally advanced NSCLC, the overall survival ranged from 13.4 to 26.8 months. A meta-analysis showed 1- and 2-year survival rates of 57% and 45%, respectively [3]. In our pre-treated cohort with locally advanced or metastatic NSCLC the median survival was 23.3 months with a 2-year survival of 46%. These interesting and surprising results confirmed Komaki et al. trial [14] where erlotinib was added to standard chemoradiation for stage III patients. They reported a very similar result to our, with a median survival of 25.8 months and 1-year OS 84%. 

Despite all the limitations of the observational nature of our study, which should be kept in mind when assigning clinical meaning to the evidence we report, adding TKIs to radiochemotherapy provided interesting results indeed. Over the next few years the clinical dilemma will be how to treat patients with locally advanced NSCLC and EGFR mutations: TKIs only, TKIs plus radiotherapy, or TKIs plus chemoradiation?

According to our experience, we can state that the addition of erlotinib to chemoradiation has a favorable safety profile and induces interestingly positive outcome. Further trials are needed to investigate the role of combined chemoradiation and TKIs or TKIs and radiation in untreated patients with EGFR mutations in order to assess the role of such treatment in locally advanced nonmetastatic NSCLC.

## Figures and Tables

**Table 1:** Patient characteristics.

	Total
*N *	60
Age (yr), median (range)	65.5 (39–83)
Sex, *N* (%)	
Male	42 (70)
Female	18 (30)
ECOG performance status, *N* (%)	
0	45 (75)
1	15 (25)
Stage, *N* (%)	
IIIA	20 (33)
IIIB	19 (32)
Relapse (mediastinal)	6 (10)
IV (oligometastatic)	15 (25)
	
Histology, *N* (%)	
Squamous cell	20 (33)
Adenocarcinoma	39 (65)
NSCLC NOS	1 (2)
Previous chemotherapy, *N* (%)	
Yes	54 (90)
No	6 (10)
Number of previous chemotherapy cycles:	
Median (range)	4 (1–12)
Response to previous chemotherapy	
PR	24 (40)
SD	28 (46)
PD	2 (4)
No previous chemo	6 (10)

Legend: PR: partial response; SD: stable disease; PD: progression disease.

**Table 2:** Treatment related toxicity.

	Grade 1-2	Grade 3-4
Hematological		
Anemia	41 (68%)	3 (5%)
White blood cell	26 (43%)	18 (30%)
Platelet	30 (50%)	7 (12%)

Nonhematological		
Esophagitis	32 (55%)	1 (2%)
Pulmonary	14 (23%)	5 (8%)
Rush	39 (65%)	4 (7%)
Diarrhea	10 (17%)	3 (5%)
Nausea	9 (15%)	—
Photophobia	5 (8%)	—
Hepatic enzymes	30 (50%)	8 (13%)
Mucositis	12 (20%)	—
